# Using the grounded theory technical approach to develop a model of Chinese physical education teachers’ functional health literacy

**DOI:** 10.3389/fpubh.2026.1794000

**Published:** 2026-04-10

**Authors:** Yunan Li, Zhihua Yin, Shanping Chen, Shanyilin Jin

**Affiliations:** 1 School of Physical Education, Xi’an Jiaotong University, Xi’an, China; 2 College of Physical Education and Health, East China Normal University, Shanghai, China; 3 Graduate School of Education, Harvard University, Cambridge, MA, United States

**Keywords:** functional health literacy, grounded theory technical approach, healthy China, PE teacher, school health education

## Abstract

**Background:**

In recent years, the frequent occurrence of public health emergencies has posed serious threats to human health, leading to a broad international consensus on the necessity of enhancing national health literacy. In response, the Chinese government has integrated health education into the national education system as a key strategic measure aimed at improving population health. The health literacy level of physical education (PE) teachers, who serve as primary agents in implementing school-based health education, is essential for ensuring the delivery of high-quality health education. Functional health literacy, as the foundational component of overall health literacy, plays a critical role in the development and enhancement of individual health competencies. In this study, we developed a structural model of functional health literacy specific to Chinese PE teachers, aiming to evaluate their own literacy levels and their contributions to school-based health education.

**Methods:**

Qualitative data were primarily collected through semi-structured interviews. A total of 16 PE teachers from 11 provinces across China mainland were purposively selected as study participants. We employed a grounded theory technical approach to analyze the data using NVIVO 20.0 qualitative analysis software.

**Results:**

The functional health literacy model for PE teachers comprises four core dimensions: reading and understanding health knowledge, numerating and calculating health data, communicating and appreciating health performance, recognizing and valuing health values. Reading and understanding health knowledge contains four categories: Health and safety emergency method, health concepts and general knowledge, sports-related health knowledge, medical health knowledge. Numerating and calculating health data include two categories: body mass index and indicators, physical fitness test data. Communicating and appreciating health performance contains three categories: health communication, health identification, health performance. Recognizing and valuing health values contains two categories: health responsibility awareness, significance and value of health.

**Conclusion:**

The proposed model offers a theoretical framework to support the professional transition of Chinese PE teachers into dual-role educators specializing in both physical education and health instruction. Applying this model with teacher education programs is expected to enhance the quality and effectiveness of health education in Chinese primary and secondary schools.

## 1 Introduction

Health literacy is defined as an individual’s capacity to obtain, comprehend, and apply basic health information and services to make informed health-related decisions (1). From a public health perspective, Nutbeam conceptualizes health literacy as a developmental construct, comprising three key dimensions: functional, interactive, and critical health literacy. Collectively, these dimensions constitute a comprehensive health literacy framework, characterized by a hierarchical progression from acquisition, to dissemination, and ultimately to decision-making. Functional health literacy encompasses fundamental literacy skills, including reading, writing, numerical computation, and communication, in order to understand health information and services (2). This construct emphasizes essential health knowledge acquisition and information processing capabilities, and it constitutes a foundational element in health literacy development. Empirical studies consistently establish significant associations between functional health literacy and health behaviors: Specifically, functional health literacy correlates with health self-awareness and chronic disease prevalence (3). Literacy levels directly mediate comprehension of dietary guidelines, consequently shaping the formation of healthy dietary habits (4). Elevated functional health literacy predicts adherence to structured health behaviors and lower rates of smoking initiation (5). Within psychosocial care contexts, patients’ functional health literacy significantly influences therapeutic outcomes (6).

Collectively, existing evidence underscores the critical role of functional health literacy in shaping personal health trajectories. Educational interventions can facilitate the development of health literacy (7), which in turn, exerts a sustained influence on health behaviors (8, 9). The curricular promotion of students’ health literacy by teachers is a primary mechanism for implementing school-based health education (10). Currently, a substantial body of scholarship acknowledges the important role of PE teachers in school health education (11). For example, Green et al. (12) reported, based on interviews with 35 secondary-school PE teachers in the United Kingdom, PE teachers have become key disseminators of health-promotion concepts and front-line practitioners of health-behavior interventions. Woods et al. (13) and Macdonald (14) highlighted the benefits of reforms to the PE curriculum for promoting health literacy and argued that instruction in strength training, health knowledge, and sport-skills teaching can improve students’ health status. In addition, research has shown that PE teachers can reduce students’ psychological difficulties, strengthen interpersonal communication, and alleviate negative affect through classroom practice (15). PE teachers’ own health literacy plays a pivotal role in the development of students’ health (16, 17). There are notable differences existed between PE specialists and general classroom teachers. When PE teachers demonstrate higher physical literacy, particularly in knowledge, self-confidence, and communication, it can play an important role in promoting students’ health (18). However, low levels of health literacy may complicate teachers’ delivery of health education (19). At the same time, observational evidence indicates that PE teachers often prioritize developing students’ physical competence and motivation while underemphasizing the knowledge and understanding components of health literacy, including the benefits of physical activity and strategies to reduce sedentary behavior (20).

In summary, PE teachers’ health literacy is pivotal to fostering healthy behaviors and advancing physical literacy among students (21). The existing literature has clarified the key role of PE teachers in school-based health education and highlighted the value of occupational health literacy in this group. However, studies indicate persistent gaps in PE teachers’ health literacy, particularly within specific knowledge domains, and confirm that teachers’ competencies directly shape student outcomes. This pattern suggests insufficient functional health literacy among current PE teachers. A considerable proportion of PE teachers lack accurate knowledge of health-related physical activity (PA) recommendations for children and adolescents. Only 7.5% could correctly identify all PA recommendations, with higher accuracy for intensity (60.5%) than for frequency (25%) or duration (37.6%) components (22). The challenge of achieving health goals within PE courses persists (12), and substantial deficiencies remain in the implementation of health-promoting instruction. This may stem from the absence of a theoretical framework for PE teachers’ functional health literacy, which hinders the establishment of pre-service and in-service training standards. Although scholars have studied in-depth research on the health service (23), communication abilities (24), and the critical health literacy of pre-service PE teachers (25), little research has attempted to construct a functional health literacy model grounded in their professional development characteristics. PE teachers’ functional health literacy, as the foundation of their health competence system, directly influences the effectiveness of daily teaching, health guidance, and student management. However, its basic structure and conceptual meaning remain insufficiently delineated. Therefore, we aim to construct a functional health literacy model for PE teachers grounded in their professional development characteristics. This model will lay the foundation for enhancing overall health literacy and will enable PE teachers to promote student health more effectively.

## 2 Materials and methods

This study adopts the grounded theory methodological framework articulated by Glaser and Strauss (26) to analyze qualitative data. Grounded theory is best understood as a methodological approach rather than a theory. Its core lies in generating theoretical models from social phenomena through the systematic analysis of qualitative data (27). Methodologically, this study is informed by constructivist epistemology, which emphasizes that knowledge emerges through interaction between researchers and participants (28), and is driven by the philosophy of pragmatism (29). The inquiry is situated within broader social, cultural, political, and technological contexts. Throughout the research process, researchers iteratively derive multi-level abstract categories from participants’ narratives of teaching and learning experiences and ultimately integrate these into a coherent conceptual framework.

**Figure 1:**
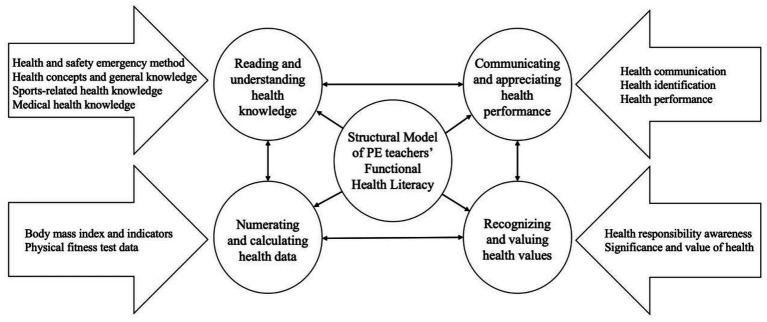
Structural model of PE teachers’ functional health literacy.

In this study, we avoided preconceived assumptions and applied a hierarchical coding strategy to derive concepts from raw data (e.g., interview transcripts). We achieved conceptual categorization and model integration through the progressive procedures of open coding, axial coding, and selective coding (see Figure 1). The grounded theory approach to investigating PE teachers’ functional health literacy is reflected in three dimensions. First, methodological congruence: PE teachers’ functional health literacy exhibits strong contextual dependence, and grounded theory techniques enable the capture of their complex practical logic within authentic work settings. Second, the advantage of theory generation: There is currently no mature theoretical framework of functional health literacy grounded in the professional development characteristics of PE teachers. In this study, we will address this gap by constructing a localized competence model through in-depth interviews with PE teachers. Third, practice oriented alignment: We draw on coding analyses of policy documents and authentic narratives from front-line PE teachers and related stakeholders, ensuring that the constructed model possesses both practical operability and policy embeddability.

### 2.1 Participants

Data for this study were obtained from interviews with 16 PE teachers from 11 provinces in China, including teachers at the primary school, junior high school, senior high school, and university. A convenience sampling method was employed, and participants were selected based on the following inclusion criteria.

Standard 1: Interviewees should be front-line or internship PE teachers.
Standard 2: Front-line PE teachers must possess at least 3 years’ teaching experience and extensive expertise in health education. Internship PE teachers should be enrolled in physical education programs accredited at Grade A or above in China, to ensure they possess sound professional teaching capabilities.
Standard 3: To ensure that study findings reflected the regional diversity, respondents were recruited from as many provinces as possible.

Charmaz in Constructing Grounded Theory emphasized that “the adequacy of a sample is determined by the depth and breadth of data needed to develop theoretical categories, not by statistical generalizations” (30). Empirical studies have likewise suggested that a sample of 10–20 participants can yield a sufficiently rich and detailed understanding of a given phenomenon (31). Based on the above considerations, the present study invited 20 experts to participate in interviews conducted between February and May 2023. 16 experts agreed to participate (see Table 1), while the remaining four declined due to time constraints.

**Table 1:** Information about the interview participants (*N* = 16).

No.	Title	Status	Province	Teaching year	Degree
1	Associate professor	University	Shanghai	10–15	Master
2	Associate professor	University	Shanghai	10–15	Master
3	Associate professor	University	Liaoning	15–20	Master
4	Associate professor	University	Liaoning	10–20	Master
5	Associate professor	University	Shanghai	20–25	Master
6	Front-line teacher	Junior High School	Guizhou	3–5	Master
7	Front-line teacher	Senior High School	Sichuan	5–10	Master
8	Front-line teacher	Senior High School	Shaanxi	3–5	Master
9	Front-line teacher	Junior High School	Fujian	3–5	Master
10	Front-line teacher	Junior High School	Henan	3–5	Master
11	Front-line teacher	Primary School	Guangdong	3–5	Master
12	Front-line teacher	Junior High School	Shanxi	3–5	Master
13	Front-line teacher	Senior High School	Shandong	3–5	Master
14	Front-line teacher	Primary School	Shanghai	5–10	Master
15	Internship teacher	Primary School	Shanghai	/	Bachelor
16	Internship teacher	Senior High School	Beijing	/	Bachelor

### 2.2 Interview

Data collection was conducted via individual in-depth interviews. Only the researcher and participant were present, and participants were informed that their privacy and confidentiality would be protected. All materials were used exclusively for research purposes. Interview questions focused on the functional health literacy of PE teachers and covered the following topics:

1. What knowledge do you have regarding health, and which theoretical aspects should PE teachers strengthen?
2. What do you think are the common health problems among PE teachers at present, and how might these be mitigated or prevented?
3. Within the prevailing “health-first” guiding ideology, what content should be included in students’ health education, and how should it be taught?
4. As a PE teacher, with which groups do you communicate about health-related issues?

Interview data including recordings from Tencent Meeting and telephone calls, which were transcribed using Feishu Yueji (web version) and then summarized, organized, and integrated. The average duration of interview materials is approximately 30 min.

### 2.3 Data analysis

This study follows the principles of the Straussian grounded theory methodology, viewing data analysis as a process of constant comparison and iteration. NVIVO 20.0 was used to code the data. Transcribed interview texts were imported in Microsoft Word format, and a bottom-up approach was employed to code the raw data (32).

#### 2.3.1 Open coding

Open coding was applied to define phenomena and concepts and to develop categories, proceeding in three steps: labeling, conceptualization, and categorization.

##### 2.3.1.1 Labeling

In this study, we aimed to identify the specific components of functional health literacy among PE teachers. Accordingly, the coding frame was defined as “functional health literacy required of PE teachers in daily life and instructional practice.” Coding was guided by the following questions: (1) Do the data pertain to PE teachers’ functional health literacy? (2) Which aspects of functional health literacy do the data reflect? (3) Do the data extend beyond the conceptual scope of functional health literacy in this study? each statement was labeled using the format of “EN (N is the numerical number) + a label statement name (e.g., E1: avoid frequently playing mahjong frequently and sitting for long periods of time), and the data were then coded (see Table 2). To avoid the coding losing its original meaning, this study tried to use the original sentences in the literature or the original words of the interviewees as tag sentences during the labeling process.

**Table 2:** Examples of raw materials labeling process.

Type	Raw materials	Labeling
1. Data source: Senior High School 2. Data number: 16 3. Collection: Individual interview	We must stretch and activate our muscles before exercising in class, and then set aside some time after class for students to stretch and relax. Many of the injuries caused by this type of exercise are due to insufficient warm-up activities and inadequate relaxation.	E105 Know to warm up, activate, stretch and relax before and after exercise.
1. Data source: University 2. Data number: 02 3. Collection: Individual interview	You need to understand the structure of the human body, and you need to be very clear about the anatomy of movement, including several major systems.	E25 Understand the anatomical knowledge of human body structure, systems, and other aspects of movement.

##### 2.3.1.2 Conceptualization

The process of conceptualization is not simply about merging labels. Instead, during the labeling process, each new label must be compared with those already generated, prompting the question: Does this represent a new phenomenon? Is it similar to or different from existing labels? Through this reflective process, labels with similar attributes are grouped together and condensed into concepts. For example, in this study, through the constant comparison of labels related to various weight management strategies, the concept of “Be able to learn scientific methods for body management or weight loss” was ultimately identified (see Table 3). This iterative process of comparison, merging, and distinction refined 47 distinct concepts from an initial set of 188 labels. The naming format for these concepts is: “DN (N is a numerical number) + concept name”.

**Table 3:** Examples of the process from raw materials to conceptualization.

Conceptualization	Labeling	Raw materials
D8 Be able to learn scientific methods for body management or weight loss	E9 Pay attention to the issue of obesity	There is a student in my class who is particularly overweight and wants to lose weight, but he does not know how to do it. I will tell him some related knowledge, such as controlling his weight through anaerobic and aerobic exercises, combined with certain strength training, and a reasonable diet.
	E31 Be able to arrange a healthy and reasonable weight loss plan	
	E122 Master the scientific exercise methods for weight loss	
	E129 Master weight control by reducing the intake of high-fat foods	
	E154 Be able to pay attention to one’s own physical management	

##### 2.3.1.3 Categorization

The final step of open coding is categorization, which involves analyzing, summarizing, and condensing the code formed by conceptualization to form and name categories. After labeling, conceptualizing, and categorizing the raw materials, this study obtained 11 categories (see Table 4), which are presented in the form of “CN (N is the numerical number) + category name”.

**Table 4:** Example of the categorization process of raw materials.

Categorization	Conceptualization	Labeling	Raw materials
C1 Health and safety emergency method	D1 Be able to learn the methods of cardiopulmonary resuscitation	E10 Can perform artificial respiration E26 Understand the knowledge of cardiac arrest E44 Proficient in using AEDs E81 Master the specific steps of cardiopulmonary resuscitation E95 Master medical emergency measures	I think most grassroots PE teachers still have the ability to perform tasks such as artificial respiration, cardiopulmonary resuscitation, emergency treatment of contusions and fractures. I have personally studied it, for example, after starting work, I obtained professional certification in this field and can master basic first aid knowledge, as well as basic dressing skills.
	D2 Be able to learn how to handle and prevent emergencies and safety accidents such as drowning, trampling, fire, heatstroke, and fainting	E52 Being able to handle heatstroke correctly E80 Master the methods of fire drills E113 Master the handling methods for stampede incidents E117 Master the Heimlich First Aid Method E118 Master the first aid methods for breathing difficulties E125 Master the handling methods for drowning E128 Master the handling methods for unexpected events in physical education class E145 Know how to deal with low blood sugar	There are also some first aid, fainting first aid, and common problems such as heatstroke that need to be learned to prevent and handle.
	D3 Be able to learn how to handle natural disasters such as earthquakes and floods	E85 Believe in the need to master knowledge of natural disasters E143 Know how to deal with natural disasters such as earthquakes E155 Know how to handle floods	In terms of safety knowledge, PE teachers may need to strengthen their skills, such as fire prevention, earthquakes, and handling of natural disasters, as well as emergency situations.

#### 2.3.2 Axial coding

During the axial coding phase, the study sought to reassemble data that had been fragmented during open coding by establishing logical relationships among categories, thereby refining main categories and enhancing their explanatory power. To support this process, the analytical trajectories recorded in memos were consulted to maintain theoretical sensitivity during category integration. Taking the identification of main category B1, “Reading and understanding health knowledge” as an example (see Table 5): in the preliminary categorization, the four categories, C1 “Health and safety emergency method,” C2 “Health concepts and general knowledge,” C3 “Sports-related health knowledge,” and C4 “Medical health knowledge” spanned considerably diverse domains, encompassing first aid skills, general knowledge, specialized theories, and medical expertise. Regarding whether they should be integrated under a single main category, the study revisited the original interview data and conducted an analysis with the aid of memos. One memo entry noted the following:

**Table 5:** Structural model of PE teachers’ functional health literacy.

Core Categories	Main Categories	Categories	Concepts
A PE teachers’ functional health literacy	B1 Reading and understanding health knowledge	C1 Health and safety emergency method	D1 Be able to learn the methods of cardiopulmonary resuscitation
			D2 Be able to learn how to handle and prevent emergencies and safety accidents such as drowning, trampling, fire, heatstroke, and fainting
			D3 Be able to learn how to handle natural disasters such as earthquakes and floods
		C2 Health concepts and general knowledge	D4 Know the harm of bad habits such as smoking, drinking, staying up late, and sitting for a long time
			D5 Ability to understand concepts and connotations related to health, sub-health, healthy behavior, health standards, etc.
			D6 Know healthy eating habits such as drinking less carbonated beverages, being picky eaters, and consuming less high calorie and high-fat foods
			D7 Know to maintain good sleep habits such as 7–8 h of sleep, going to bed early, and waking up early
			D8 Be able to learn scientific methods for body management or weight loss
			D9 Master certain sexual health education knowledge
		C3 Sports-related health knowledge	D10 Know that reasonable and scientific exercise can promote health
			D11 Master good exercise habits such as warm-up and relaxation before and after exercise, protection and assistance during exercise, and avoiding taking cold showers or drinking a lot of water after exercise
			D12 Can understand books such as “Sports Anatomy”, “Nutrition”, and “Physical Education and Health Curriculum Standards”
			D13 Master the prevention and treatment methods for sports injuries such as sprains, contusions, dislocations, abrasions, and strains
			D14 Be able to learn professional sports-related health knowledge such as exercise-induced abdominal pain, muscle performance enhancement, and exercise rehabilitation methods
		C4 Medical health knowledge	D15 Be able to learn the basic knowledge and preventive measures of common respiratory tract (COVID-19, influenza, chicken pox, etc.) and digestive tract (hand foot mouth disease, red eye disease, etc.) infectious diseases
			D16 Can understand drug instructions
			D17 Can understand oral health knowledge
			D18 Understand the characteristics of diseases such as asthma, tuberculosis, myocarditis, etc.
			D19 Can understand medical appointment forms, medical records, and laboratory test reports, etc.
	B2 Numerating and calculating health data	C5 Body mass index and indicators	D20 Ability to recognize and calculate common indices such as BMI, body fat percentage, and basal metabolism
			D21 The standard range for normal blood pressure is between 90 ~ 140/60 ~ 90 mmHg (systolic blood pressure range is 90 ~ 140 mmHg, diastolic blood pressure range is 60 ~ 90 mmHg)
			D22 Know that the normal temperature range for the human body is 36 °C -37 °C
			D23 Know that the normal pulse range of the human body is 60–100 beats per minute
			D24 The calculation method for knowing the target heart rate is 170-age
		C6 Physical fitness test data	D25 Be able to correctly calculate the sum of the product of the scores and weights of each individual indicator in the national physical fitness test
			D26 Be able to correctly classify test scores into four corresponding evaluation levels, namely “excellent”, “good”, “passing”, and “failing”
			D27 Can understand the scoring table for each individual indicator in the national physical fitness testing standards
			D28 Can understand the weight distribution table of individual indicators in the national physical fitness testing standards
	B3 Communicating and appreciating health performance	C7 Health communication	D29 Be able to acquire medical and health knowledge through communication with doctors and others
			D30 Be able to acquire sports-related health knowledge through communication and exchange with professional athletes, experienced teachers, or colleagues
			D31 Be able to promote mental health through communication with psychological counselors and other professionals
		C8 Health identification	D32 Be able to determine whether oneself and others are in a healthy state
			D33 Can determine whether dietary and sleep habits belong to healthy behaviors
			D34 Can determine the type of sports injury
			D35 Can identify what junk food or harmful food is
			D36 Ability to identify drugs
		C9 Health performance	D37 Skilled at discovering beauty and enjoying life
			D38 Full of interest in physical exercise
			D39 Capable of self-regulation and stress relief
	B4 Recognizing and valuing health values	C10 Health responsibility awareness	D40 Believe that it is their responsibility to popularize and disseminate health knowledge
			D41 Recognize that maintaining one’s own and others’ health is a responsibility
			D42 Have the awareness of actively learning health knowledge
			D43 Respect and acknowledge individual health differences
		C11 Significance and value of health	D44 The important role of identifying with a healthy lifestyle and behavioral habits
			D45 Recognize health as the foundation and prerequisite for personal development and family happiness
			D46 Clarify the practical and long-term significance of health to China
			D47 Believe that health education is very important

*When multiple teachers discussed their methods of acquiring health knowledge during the interviews, their accounts were highly similar, such as reading textbooks, attending lectures, and searching for information online. Although the specific content they referred to varied, the essence of the described behavior was consistent: receiving and understanding health-related information from external sources through reading, listening, and observing. That is, regardless of the content, what they were doing essentially involved “reading and comprehending.”*

This suggested to me that the basis for categorization should not be “what was read,” but rather “what was being done.” Based on this analysis, the study merged C1 through C4 into the main category B1. This refining process embodies the pursuit of “substantive connections” between categories during the axial coding phase, that is, transcending the surface-level content to focus on the commonality in cognitive operations.

We summarized four main categories, presented in the form of “BN (N is a numerical number) + category name”, namely B1 reading and understanding health knowledge, B2 numerating and calculating health data, B3 communicating and appreciating health performance, and B4 recognizing and valuing health values.

#### 2.3.3 Selective coding

Selective coding is the synthesis phase, in which a core category that systematically links all main categories is identified, guided by memos throughout the study. Through memo review and constant comparison of the four main categories, a central storyline emerged. The analysis then returned to all categories, concepts, and labels to verify whether this hypothesized core adequately accounted for the data. Through this iterative process, the structural model of PE teachers’ functional health literacy was developed.

#### 2.3.4 Theoretical saturation test

To ensure that the functional health literacy structure model for PE teachers captured all relevant aspects, theoretical saturation testing was conducted (33). This study employed two validation tests. First, to ensure coding reliability, we randomly selected five interview transcripts. Two researchers independently coded them, and the intercoder reliability was calculated and compared with the initial coding results. Second, 80% of the original data were used for the analysis, and the remaining 20% and other related materials were used for theoretical saturation testing. Analysis of the remaining 20% of the data using NVIVO 20.0 revealed no new nodes, indicating that data saturation had been reached. Therefore, the structural model of functional health literacy for PE teachers can be considered both reliable and empirically validated.

## 3 Result

Drawing on the social and cultural context of China, the construction process of the functional health literacy structure model for PE teachers is as follows: labels → concepts → categories → main categories → core categories. The model consists of 4 main categories, 11 categories, 47 concepts, and 188 labels (Table 5, Figure 1).

### 3.1 Reading and understanding health knowledge

“Reading and understanding health knowledge” refers to the integration of individuals’ basic cultural literacy such as reading and understanding with health knowledge. It is a prerequisite for PE teachers to master health skills, deliver health teaching, and provide health services. It covers four aspects: health and safety emergency method, health concepts and general knowledge, sports-related health knowledge, and medical health knowledge.

#### 3.1.1 Health and safety emergency method

“Health and safety emergency method” refers to the procedures and measures used in emergencies, including cardiopulmonary resuscitation, fire stampedes, sudden events such as heatstroke and fainting, and common natural disasters. As the primary organizers of PE courses and campus activities, PE teachers must master health and safety emergency method to ensure student safety and smooth operation of course activities.

During the interview, an interviewee mentioned this aspect: “Students are also prone to sudden cardiac arrest while running 800 meters, so it is important to learn how to use AEDs well and have a grasp of the golden time for artificial respiration and cardiopulmonary resuscitation.” (Interviewee 16).

#### 3.1.2 Health concepts and general knowledge

“Health concepts and general knowledge” refers to the basic knowledge relevant to daily life, including unhealthy behavior habits, nutrition and diet knowledge, sleep knowledge, weight management, and ways of acquiring health knowledge. This knowledge is a prerequisite for PE teachers to maintain their own health, deliver health education, and provide health services.

Some respondents state that “PE teachers should pay attention to a reasonable diet, consume sufficient vitamins and proteins every day, eat more fresh vegetables and fruits. They should also practice good hygiene, such as washing hands frequently, rinsing mouth after meals, brushing teeth, etc. In an addition, they should ensure adequate sleep and, if possible, take a daily nap.” (Interviewee 15).

#### 3.1.3 Sports-related health knowledge

“Sports-related health knowledge” refers to systematic and integrated knowledge related to physical exercise, sports science, and health education. For PE teachers, mastery of this knowledge constitutes a core competency that directly affects student safety, promotes scientific rigor, and fosters lifelong healthy exercise habits within sports and health curricula. It mainly includes understanding the health benefits of exercise, cultivating good exercise habits, the ability to interpret professional literature, the prevention and treatment of sports injuries, and advanced professional skills such as sports rehabilitation and methods for enhancing muscle performance.

One respondent stated, “In the new era, PE teachers must first master professional theoretical knowledge and solid professional skills. Before teaching, I will perform some warm-up activities myself. While modeling proper self-protection, I can demonstrate more accurately and effectively to students, thereby achieving better teaching results.” (Interviewee 1).

#### 3.1.4 Medical health knowledge

“Medical health knowledge” refers to systematic knowledge related to disease prevention and healthcare. Within the national strategy of “integration of sports and medicine”, PE teachers, who play an important participant in grassroots sports and medicine integration practice, must possess necessary medical knowledge and health literacy. However, given the professional background and practical needs of PE teachers, their knowledge reserves should remain at a basic and applied levels without requiring the depth and breadth expected of the medical professionals. Accordingly, this study defines the scope of medical and health knowledge that Chinese PE teachers should master two following core dimensions: (1) identifying and preventing common diseases, and (2) understanding and applying basic medical information. This definition aligns with the practical responsibilities of PE teachers and supports the integration of sports and medicine in education.

As stated by the interviewee: “As a PE teacher, I have a good understanding of common illnesses such as colds, fever, diarrhea, and headaches, as well as other common illnesses that my family members may have, such as hypertension and hyperlipidemia. I also teach my students about these topics.” (Interviewee 3).

### 3.2 Numerating and calculating health data

“Numerating and calculating health data” refers to the integration of individuals’ basic cultural literacy such as recognizing and calculating numbers with health-related data and various physical indicators. It provides the foundation for individuals to interpreting and applying health data, such as “Body mass index and indicators” and “Physical fitness test data”.

#### 3.2.1 Body mass index and indicators

“Body mass index and indicators” refers to quantitative parameters that can objectively reflect an individual’s physiological status and health level. These include the normal reference range of basic physiological indicators (such as body temperature, blood pressure, etc.), as well as the calculation method of derived health indices (such as body mass index BMI, body fat percentage, target heart rate, etc.). These indices and indicators are closely related to general health, sports health, and medical health knowledge. They also highlight the need for the basic cultural literacy of people in functional health literacy-mathematical and physical abilities. Specifically, PE teachers need to have the ability to accurately identify, calculate, and interpret these indicators. This requirement reflects the integration of sports and medicine in physical education and underscores the importance of basic subject literacy (such as mathematical operations and biological knowledge) in the contemporary PE teacher training system.

If an interviewee says, “I think PE teachers need to understand and master BMI, blood sugar and blood pressure, and basal metabolic rate, especially how to calculate BMI, and also teach students to help them establish relevant concepts.” (Interviewee 6).

#### 3.2.2 Physical fitness test data

“Physical fitness test data” refers to the core indicators used in school sports work to systematically evaluate students’ physical fitness and health levels. The test content includes physical shape, physical function, and physical fitness. This indicator mainly emphasizes the mastery of the weight allocation, score conversion methods, and grading standards for each test item within the national physical fitness testing standards. Under the current education system in China, both compulsory education and higher education include student physical fitness and health testing as mandatory assessment items. As the primary implementer of testing, PE teachers must be fully familiar with the measurement norms of the national “Student Physical Fitness and Health Standards”. Their ability to process physical fitness test data directly affects the accuracy and reliability of national student physical fitness and health monitoring data.

Some respondents believe that “PE teachers are often responsible for tasks such as inputting and calculating grades during their final work, such as conducting physical fitness tests for students in public physical education classes, which require converting and scoring their data. As PE teachers, we need to have the ability to accurately identify and calculate this data.” (Interviewee 1).

### 3.3 Communicating and appreciating health performance

“Communicating and appreciating health performance” refers to the integration of individuals’ basic cultural literacy such as communication, identification, and appreciation with the content and presentation forms of health display. It is an important means of improving individual health and enhancing participation in health culture, comprising three dimensions: health communication, health identification, and health performance.

#### 3.3.1 Health communication

Under the concept of functional health literacy, “Health communication” specifically refers to the process by which individuals acquire health knowledge and promote their own health through communication. The group of PE teachers mainly manifests as interactions with doctors, professional athletes, colleagues, and psychological counselors. It is worth noting that the concept of “health communication” does not belong to the category of interactive health literacy. Its essence is a one-way behavior of acquiring health knowledge, with the core purpose of improving one’s own health, rather than disseminating health knowledge. PE teachers have access to certain professional resources and networks. In daily work, targeted health consultations can be conducted with professional personnel such as school doctors and psychological counselors. At the same time, a sustainable health knowledge acquisition system can be established by drawing on the expertise of multidisciplinary teaching team on campus (e.g., biology, psychology and related subjects).

As stated by the interviewee, “When I feel pain in a joint, I consult the sports anatomy teacher at the college. Through these discussions, I have learned the specific cause of such pain. When we don’t have classes, we also have many teachers together, some who teach physiology and some who teach anatomy. I can learn more professional health knowledge from them.” (Interviewee 4).

#### 3.3.2 Health identification

“Health identification” refers to the ability to use one’s own knowledge of health to judge whether things, behaviors, and habits are healthy. It is a prerequisite for PE teachers to timely correct and prevent health problems and provide correct health services. Its essence is the integration of professional health knowledge and basic cultural judgment, which is a composite literacy.

An interviewee said, “PE teachers need to be able to judge what kind of health status they or others are currently in. For example, when I am in physical education class, I may observe that some students appear tired from staying up late the previous night and are therefore not fit for exervice. I will actively ask them and tell them what kind of exercise this state is not suitable for. This basic discernment ability is what PE teachers should possess.” (Interviewee 2).

#### 3.3.3 Health performance

“Health performance” refers to the explicit characteristics exhibited by individuals that meet health standards, and is the most direct manifestation of whether an individual is healthy for PE teachers, health performance encompasses three aspects: enjoying life, sustaining interest in sports, and regulating psychological well-being. In the context of school education, PE teachers with good health performance can not only promote the development of students’ health behaviors through demonstration effects, but also create a “health attraction” that encourages students and others to seek health advice from them.

An interviewee stated, “PE teachers must first be physically healthy in order to be able to teach by example. If a PE teacher’s physical health does not meet proper good standards, it will be difficult for them to teach persuasively. In addition, PE teachers must also be psychologically healthy, as their professional ethics and teaching style can be reflected through their mental health. If a PE teacher’s mental health is very healthy, sunny, optimistic, and positive, then they can better exert their infectious power in teaching and influence more people.” (Interviewee 4).

### 3.4 Recognizing and valuing health values

“Recognizing and valuing health values” refers to an individual’s affirmation of the meaning and value of health at the cognitive level, emphasizing the accurate understanding of health promotion, health learning, and health education. It serves as an inherent motivation for continuously improving health literacy and providing health services. It mainly includes two core dimensions: “Health responsibility awareness” and “Significance and value of health.”

#### 3.4.1 Health responsibility awareness

“Health responsibility awareness” refers to the sense of responsibility towards one’s own and others’ health. It includes four key dimensions: (1) health service responsibility, the cognitive responsibility to provide professional health guidance; (2) health maintenance responsibility, actively participating in health management; (3) health learning responsibility, maintaining a proactive attitude towards continuously updating health knowledge; and (4) responsibility for health disparities, social responsibility to pay attention to and address health inequalities.

One interviewee explained as follows: “In addition to popularizing knowledge about cardiopulmonary resuscitation and first aid, I think PE teachers should also take responsibility for teaching student health knowledge such methods for managing sports injuries.” (Interviewee 6).

#### 3.4.2 Significance and value of health

“Significance and value of health” refers to the affirmation and recognition of the multidimensional value of health, including value recognition of healthy behavior, personal value recognition of health, social value recognition of health, and educational value recognition of health. As key actors in promoting health, PE teachers must transform these cognitive dimensions into professional practice through the process of internalizing values.

As stated by the interviewee, “I regularly explain the importance of health in today’s society to my students, and I incorporate some health concepts into every class I teach.” (Interviewee 3).

## 4 Discussion

### 4.1 Higher standards and professional characteristics of functional health literacy for PE teachers

In terms of specific connotations, the functional health literacy of PE teachers demands greater professional competencies and higher levels of expertise compared to other teachers. PE teachers are increasingly recognized for their role in delivering holistic health education, not just focusing on physical activity but also addressing emotional and social well-being (34). This indicates that PE teachers’ functional health literacy must encompass broader knowledge, stronger professional skills, and stricter standard.

In the fields of “Health and safety emergency method” “Health concepts and general knowledge” “Sports-related health knowledge” “Medical health knowledge” as well as “Body mass index and indicators”, other teachers only need to master basic content to meet their personal health needs, whereas PE teachers must develop a deeper and more comprehensive mastery of the knowledge, continuously updating their expertise to support effective teaching and practice. For example, in the face of emergency situations such as sudden cardiac arrest of students, PE teachers need to be able to accurately implement emergency measures such as cardiopulmonary resuscitation. At the level of “Health responsibility awareness” and “Significance and value of health”, PE teachers place greater emphasis on the recognition health services and communication, respect for health differences, multidimensional perspectives on health, and the value of health education. The ability to calculate and analyze physical fitness test data is a unique competency required of PE teachers and is an essential for the validity and accuracy of physical fitness assessment. As for “Health communication” “Health identification” and “Health performance,” PE teachers must actively acquire health knowledge from diverse sources, demonstrate positive health behaviors, and exhibit more heightened sensitivity in identifying health-related behaviors compared to non-specialists. These requirements exceed the standards expected of teachers in other subject areas.

### 4.2 The important value of the structural model of functional health literacy for PE teachers

At the level of personal development, the structural model constructed in this study provides a fundamental theoretical reference for improving the overall health literacy level and health education ability of PE teachers. “The Notice of the Ministry of Education on Strengthening the Construction of the PE Teacher Team in Primary and Secondary Schools in the New Era” (January 2015) emphasized the need to improve the professional literacy level in health knowledge, identifying health literacy as a core element of their professional development. However, research from countries including China (35), Iran (36), Sri Lanka (37), and Germany (38) indicates that PE teachers’ health literacy remains inadequate, limiting their ability to fulfill the responsibility of school health education. The functional health literacy structure model for PE teachers constructed in this study involves key dimensions such as health knowledge, health concepts, health behaviors, health management, and health communication. It is closely aligned with PE teachers’ health literacy and health education competencies (such as emergency response, health communication, and health education teaching). It is helpful to improve the overall teaching ability of PE teachers, so as to better promote the development of students’ physical literacy and improve the teaching effect (39).

At the level of curriculum implementation, the functional health literacy structure model of PE teachers is in line with the current direction of curriculum reform that deeply integrates physical education and health education (40). Since the founding of the People’s Republic of China, the PE curriculum standards have been undergone multiple revisions in the area of health education. These include adding a teaching module on “basic knowledge of physical health”, emphasizing on cultivation of healthy behavioral qualities in students (41), and proposing a “three-dimensional health concept”. The guiding ideology of “health first” has become firmly embedded in educational policy and practice (42). The “Compulsory Education Physical Education and Health Curriculum Standards” (2022 Edition) released during China’s eighth physical education and health curriculum reform includes “health education” as one of the five major contents, consolidating the health attributes of physical education curriculum. The “Sports and Health” course sets higher requirements for integrating physical education and health education. However, there is still an imbalance between health education and physical education teaching in China’s current physical education and health curriculum. To effectively integrate the two, it is urgent to enhance the “life oriented” dimension of health education, ensuring that its content reflects students’ daily life, developmental needs, and social realities. In this regard, Australia’s health education model offers a valuable reference, as 50% of its curriculum standards (across six key areas) are directly linked to health education and highly emphasize life-oriented practices such as food nutrition recognition, safe use of drugs, and the health benefits of physical activity, while closely integrating with national health guidelines and real-life scenarios (43). Grounded in these insights, the structural model developed in this study encompasses critical aspects such as emergency response, reasonable exercise, nutritious diet, disease prevention, physical fitness monitoring, health judgment, and health appreciation. By emphasizing the practicality and daily relevance of health knowledge, the model provides a systematic framework for supporting the coordinated development of physical education and health education.

At the policy level, the model can facilitate the implementation of China’s health strategy. The “Healthy China 2030” Plan Outline emphasizes integrating health education into the national education system and establishing it as a crucial component of quality education across all stages. The health literacy of PE teachers is a prerequisite for ensuring the effectiveness of health education and directly influences the successful implementation of health strategies. The model constructed in this study clarifies the core elements of functional health literacy for PE teachers, providing ability guidance and evaluation criteria for policy implementation. For example, the model emphasizes the ability to understand and apply data, requiring PE teachers to accurately calculate and interpret indicators such as BMI, body fat percentage, and national physical fitness test scores, providing a data basis for scientific evaluation and health intervention of students’ physical fitness; Regarding the communication and appreciation of health performance, teachers are required to obtain a wider range of health knowledge resources through communication with doctors, psychological counselors, athletes, and other parties, and have the ability to identify health behaviors, dietary safety, and drug hazards, in order to ensure the cultivation of students’ health behaviors. This model transforms macro policy requirements into specific teacher competency indicators, facilitating he transition of health strategies from policy documents to educational practices, and providing a clear, actionable pathway for the construction of a Healthy China.

### 4.3 Practical path for enhancing the health literacy of Chinese PE teachers

Currently, programs to develop health literacy among PE teachers are offered in countries around the world. In Australia, a professional development program improved PE teachers’ knowledge and understanding of health literacy levels and implementation strategies (44). The personalized nature of this program was identified as a key strength, improving teachers’ knowledge and understanding of health literacy levels. Similarly, in other countries, professional development for both generalist and specialist PE teachers is recognized as essential for nurturing physical literacy within teaching practice (45). In the future, there is also a need for health literacy improvement programs for PE teachers in China.

#### 4.3.1 Optimize the cultivation mode of pre-service PE teachers

To enhance the health literacy of PE teachers, China should refine the training mode of pre-service PE teachers’ health education competencies, while simultaneously fostering both health skills and cultural literacy alongside the development of physical education expertise. At the level of cultivating health competencies, universities should offer specialized courses, such as health education methodology, to support the development of future teachers’ health teaching skills. At the level of promoting cultural literacy, the curriculum’s teaching model should be optimized to focus on enhancing students’ Chinese reading comprehension, mathematical application, and artistic appreciation and expression skills in physical education programs. For example, university teachers can use case teaching method to analyze health science popularization texts, arrange practical activities for students to analyze physical fitness test data, providing the most basic literacy guarantee for their independent learning of health knowledge, improving health literacy, and efficient health teaching practice after employment. At the level of shaping attitudes and values, methods such as role model promotion and case study discussions can be employed to strengthen pre-service teachers’ sense of professional responsibility and awareness of serving as health role models. For instance, physical education students could be organized to participate in community health volunteer services, allowing them to internalize health values through practical experience.

#### 4.3.2 Refine the professional standards for PE teachers

Relevant institutions and government agencies should further refine the professional standards for PE teachers (46) to enhance their understanding and application of health literacy knowledge (44). As PE teachers transition into physical education and health teachers, existing documents such as the “Professional Standards for Middle School Teachers (Trial)” no longer adequately address the professional development needs of PE teachers in the contemporary context, particularly in relation to health literacy dimensions, which exhibit significant gaps. By integrating the model developed in this research, the following elements can be systematically supplemented into the current standards, thereby connecting macro-level literacy dimensions with concrete and actionable assessment measures, and providing a well-defined foundation for the training, qualification, and evaluation of PE teachers (Table 6).

**Table 6:** Example of PE teachers’ professional standards based on model.

Main categories	Standard	Content example	Example of assessment action
Reading and understanding health knowledge	Health knowledge standard	Basic medical knowledge	Assess medical knowledge through multiple-choice questions Summarize the main points of health popularization articles
Numerating and calculating health data	Health standard	Health data collection	Calculate an individual’s BMI and explain its significance Analyzing and interpreting physical fitness test data
Communicating and appreciating health performance	Health performance standard	Own health level	Students evaluate teachers’ daily health conditions Teachers’ self-assessment of their health status
Recognizing and valuing health values	Health attitude standard	Health values	Assessing health values through questionnaires Observe the attitude and demonstration behavior in activities

#### 4.3.3 Improve the professional organization for the development of PE teachers

Weber et al. (47) assert that professionalism is a social structure that runs parallel to government, legislation, and market regulation, playing a crucial role in coordinating and resolving conflicts of social interests. As a product of professionalism, teacher professional organizations contribute to educational reform by enhancing teachers’ professional development and quality through providing training, strengthening industry oversight, and participating in standard setting. Furthermore, the interview process of the study revealed certain occupational health issues among current Chinese PE teachers. Therefore, to ensure the health status, which is essential for the development and enhancement of PE teachers’ health literacy (48), professional organizations must also establish mechanisms to safeguard the occupational health of PE teachers, pay attention to the health burden of PE teachers, and give them more health care.

### 4.4 Theoretical basis for the structural model of functional health literacy of PE teachers

The functional health literacy structure model for PE teachers is a fusion of relevant scientific theories, including “Knowledge–Attitudes–Beliefs–Practice (KABP) Theory”, “Health Belief Model (HBM)”, and “Health-Promoting Schools (HPS) Theory”. These theories align with the conceptual framework of the functional health literacy structural system for PE teachers.

This study primarily draws on the KABP Theory (49), which posits that health knowledge is a prerequisite for behavior change, with attitude and belief serving as key mediating variables, and health behavior as the ultimate outcome. The functional health literacy of PE teachers is categorized into four main categories, corresponding to the three elements of “knowledge” “belief” “behavior” in KABP Theory: at the knowledge level, it involves knowledge content in multiple fields such as sports-related health knowledge, reflected in the two main categories of “Reading and understanding health knowledge” and “Numerating and calculating health data”, highlighting the understanding, recognition, and calculation abilities of PE teachers towards health information. At the belief level, it is embodied in the category of “recognizing and valuing health values”, emphasizing the recognition of health values and the responsibility of health education by PE teachers, which is the psychological motivation for promoting the sustainable development of their health behaviors. At the behavioral level, it centers on the main category of “Communicating and appreciating health performance”, manifested as the ability of PE teachers to communicate health knowledge, judge health behaviors, and demonstrate health performance in both teaching and life, representing the external manifestation of functional health literacy.

On this basis, the HBM (50) further enriches and deepens the structural logic of KABP Theory. HBM posits that the occurrence of individual health behaviors depends on their perception of health threats, evaluation of behavioral benefits and barriers, and confidence in self-efficacy. The formation of these core cognitions is grounded in functional health literacy: for example, if teachers do not possess first aid knowledge, sports injury coping skills, or the ability to analyze health data, it is difficult to form sufficient risk perception and behavioral confidence. Therefore, functional health literacy underpins the cognitive assessments upon which HBM relies and serves as the foundation for its cognitive motivation mechanism. The two together form a logical closed loop of healthy behavior from “able to do” to “willing to do”, achieving a layered promotion of “knowledge belief behavior”.

HPS Theory (51) emphasizes incorporating health into the overall development strategy of the school, improving the health level of all members through curriculum integration, environmental optimization, and teacher strengthening. This theory advocates that educators should move beyond an exclusive focus on individual health behaviors and adopt systematic, collective health interventions. Therefore, PE teachers not only need to possess the basic literacy of maintaining their own health, but also need to have the comprehensive ability to guide students to form healthy behaviors and participate in the formulation and implementation of campus health policies. For example, in the functional health literacy structure system, dimensions such as “Health and safety emergency method” and “Physical fitness test data” are the literacy guarantees for PE teachers to transition from individual knowledge practitioners to collective health leaders in campus health promotion.

## 5 Conclusion

This study employed grounded theory technical approach to develop a structural model of functional health literacy among Chinese PE teachers. This model comprises four dimensions (reading and understanding health knowledge, numerating and calculating health data, communicating and appreciating health performance, recognizing and valuing health values), forming 11 categories based on 47 concepts and 188 labels. In these categories, “Reading and understanding health knowledge” includes four components: health and safety emergency method, health concepts and general knowledge, sports-related health knowledge, medical health knowledge. “Numerating and calculating health data” include two categories: body mass index and indicators, physical fitness test data. “Communicating and appreciating health performance” includes three categories: health communication, health identification, health performance. “Recognizing and valuing health values” includes two categories: health responsibility awareness, significance and value of health. The results of theoretical saturation and consistency tests indicate that the model demonstrates strong reliability and validity, and can comprehensively explain the functional health literacy of Chinese PE teachers.

## 6 Limitations and future research

This study applies grounded theory technical approach and adheres a scientific research procedure to develop a valuable functional health literacy structural model for future PE teachers. However, there are some limitations to the research, and the following aspects warrant further exploration in the future.

First, this study adopted purposive sampling to select 16 PE teachers as interview participants. It should be acknowledged that the sample size may limit the generalizability of the findings to broader educational contexts. Furthermore, the sample was restricted to PE teachers, excluding the perspectives of public health practitioners (e.g., university health education instructors, physicians), which may limit a comprehensive understanding of PE teachers’ functional health literacy. Future studies could consider broadening the sample to include experts from multiple disciplines, thereby enhancing the diversity and representativeness of the findings.

Second, this study concentrates on the functional health literacy of Chinese PE teachers, without considering international development. Therefore, the functional health literacy structure model of PE teachers developed in this study may not be applicable to global PE teacher education programs. In future studies, researchers in the field of global PE teacher education could follow the procedures outlined in this study and apply grounded theory to construct functional health literacy structural models for PE teachers that are applicable across diverse national contexts.

Third, in terms of sample composition, the relatively small number of pre-service PE teachers included in this study may have limited the capture of perspectives specific to the pre-service stage. Additionally, differences were observed between primary/secondary school and university PE teachers in their understanding of functional health literacy, suggesting that the requirements for PE teachers’ health literacy may be hierarchical and stage-specific across different educational levels. Future research could examine the differentiated needs of pre-service, primary/secondary, and university PE teachers to develop more targeted structural models of literacy.

## Data Availability

The raw data supporting the conclusions of this article will be made available by the authors, without undue reservation.
