# Supplementary material for: Using the grounded theory technical approach to develop a model of Chinese physical education teachers’ functional health literacy
Source: Front Public Health. 2026 Apr 10;14:1794000. doi: 10.3389/fpubh.2026.1794000 (PMC13106416; doi:10.3389/fpubh.2026.1794000)
Supplement: Supplementary file 1 [file Table_1.docx]

**Structure of Functional Health Literacy for PE Teachers: Interview Guide**

Dear Sir or Madam,

In response to the Healthy China strategy, national authorities have progressively intensified their emphasis on health education in schools, as reflected in policy initiatives such as the Healthy China 2030 plan. Guided by the principle of “health first,” physical education and health curricula have become essential elements of school-based health education.

As the principal agents of school physical education, the functional health literacy of physical education teachers is of paramount importance, as it directly influences the development of students’ health literacy. This study primarily investigates the structure of functional health literacy among physical education teachers, with the objective of developing an evaluation framework for this construct. The resulting framework is expected to provide a more scientific basis and practical guidance for advancing health education within school physical education programmes.

Because you are currently employed in the field of physical education, you are cordially invited to participate in an interview regarding the structure of functional health literacy among physical education teachers. The interview will be conducted primarily in a question and answer format. All data collected during the interview will be treated as strictly confidential and will have no adverse effects on you.

To ensure the validity of the interview, we kindly request that you respond to the questions honestly. Your participation will make an important contribution to improving the theoretical and practical understanding of health education in schools.

Thank you for your cooperation.

Yunan Li

School of Physical Education and Health, East China Normal University

Contact: 375762286@qq.com, 17741881887

December 2022

**1. What knowledge do you have regarding health, and which theoretical aspects should PE teachers strengthen?**

E.g. (1) How do you conceptualise the notion of “health”? (2) In your understanding, how are “physical health,” “mental health,” and “social well-being” defined and distinguished? (3) What core knowledge do you possess concerning health-related domains (such as nutrition, sleep, medical care, disease prevention, safety, and exercise)? (4) From your perspective, what essential criteria should be met to ensure the overall health of physical education teachers? (5) What fundamental methods of health-related measurement and calculation are you familiar with (for example, body mass index, blood glucose and blood pressure monitoring, or basal metabolic rate)? (6) Which strategies or methods for the management of sports injuries should physical education teachers be able to master?

**2. What do you think are the common health problems among PE teachers at present, and how might these be mitigated or prevented?**

E.g. (1) What common health concerns or challenges do you and your colleagues currently encounter in your professional or personal lives? (2) In your view, what measures or strategies could be adopted to improve these health conditions or to prevent such problems from arising? (3) Through which channels or sources do you usually obtain health-related information, and how might these contribute to enhancing your personal well-being?

**3. Within the prevailing “health-first” guiding ideology, what content should be included in students' health education, and how should it be taught?**

E.g. (1) How do you interpret the concept of ‘healthy behaviour’ within the three core competencies, and how do you cultivate the development of healthy behaviours among your students in your teaching practice? (2) What health concepts and awareness do you instil in your students?

**4. As a PE teacher, with which groups do you communicate about health-related issues?**

E.g. Communicating medical conditions, providing/obtaining health information, and exchanging health education teaching capabilities.
